# GDF11 administration does not extend lifespan in a mouse model of premature aging

**DOI:** 10.18632/oncotarget.11096

**Published:** 2016-08-05

**Authors:** Sandra Freitas-Rodríguez, Francisco Rodríguez, Alicia R. Folgueras

**Affiliations:** ^1^ Departamento de Bioquímica y Biología Molecular, Facultad de Medicina, Instituto Universitario de Oncología del Principado de Asturias (IUOPA), Universidad de Oviedo, Oviedo, Spain

**Keywords:** progeria, accelerated aging, GDF11, longevity, Gerotarget

## Abstract

GDF11 has recently emerged as a powerful anti-aging candidate, found in young blood, capable of rejuvenating a number of aged tissues, such as heart, skeletal muscle and brain. However, recent reports have shown contradictory data questioning its capacity to reverse age-related tissue dysfunction. The availability of a mouse model of accelerated aging, which shares most of the features occurring in physiological aging, gives us an excellent opportunity to test *in vivo* therapies aimed at extending lifespan both in pathological and normal aging. On this basis, we wondered whether the proposed anti-aging functions of GDF11 would have an overall effect on longevity. We first confirmed the existence of a reduction in GDF11/8 levels in our mouse model of accelerated aging compared with wild-type littermates. However, we show herein that GDF11 daily administration does not extend lifespan of premature-aged mice.

## INTRODUCTION

The existence of “rejuvenating” factors in young blood capable of improving the function of aging stem cells was first demonstrated in 2005 by the group of Tom Rando [1]. A decade after this seminal contribution, the new wave of studies has been on the search for those circulating regulatory molecules that can restore the regenerative function of old stem cells and reverse aging [2-4]. Among several cell-extrinsic factors and metabolites identified to date, GDF11 has been found to be one of the most powerful anti-aging candidates. Thus, it has been shown that GDF11 levels in blood decline with age, and that its supplementation to reach young physiological range in old mice improved the features and function of a number of age-deteriorated tissues, including heart, skeletal muscle and brain [5-8].

Many of the symptoms associated with normal aging have also been reported in human accelerated aging syndromes, such as Hutchison-Guilford progeria, a devastating disease caused by alterations in the nuclear envelop architecture [9-11]. Those include skin wrinkling, hair loss, muscle atrophy, osteoporosis and a premature cardiovascular disease, which is responsible for the death of the patients in the childhood due to myocardial infarction or stroke [12]. The generation of mouse models that phenocopy most of the features of this syndrome, such as mice deficient in the metalloproteinase Zmpste24, which is required for nuclear lamin A maturation, represents useful tools for studying not only the mechanisms underlying this disease, but also those common to normal aging process [12-14].

Our previous studies with a mosaic mouse model in which Zmpste24-deficient cells coexist with Zmpste24-proficient cells in similar proportion showed a complete correction of the progeroid phenotype [15]. These results demonstrated the relevance of cell-extrinsic mechanisms in the establishment of this pathology and suggested that a therapeutic approach based on the administration of key systemic factors may be an avenue to improve the progression of this disease. On this basis, and given the proposed anti-aging functions of GDF11, we analyze herein the *in vivo* effect of GDF11 administration on the lifespan of premature-aged mice.

## RESULTS

**Figure 1:**
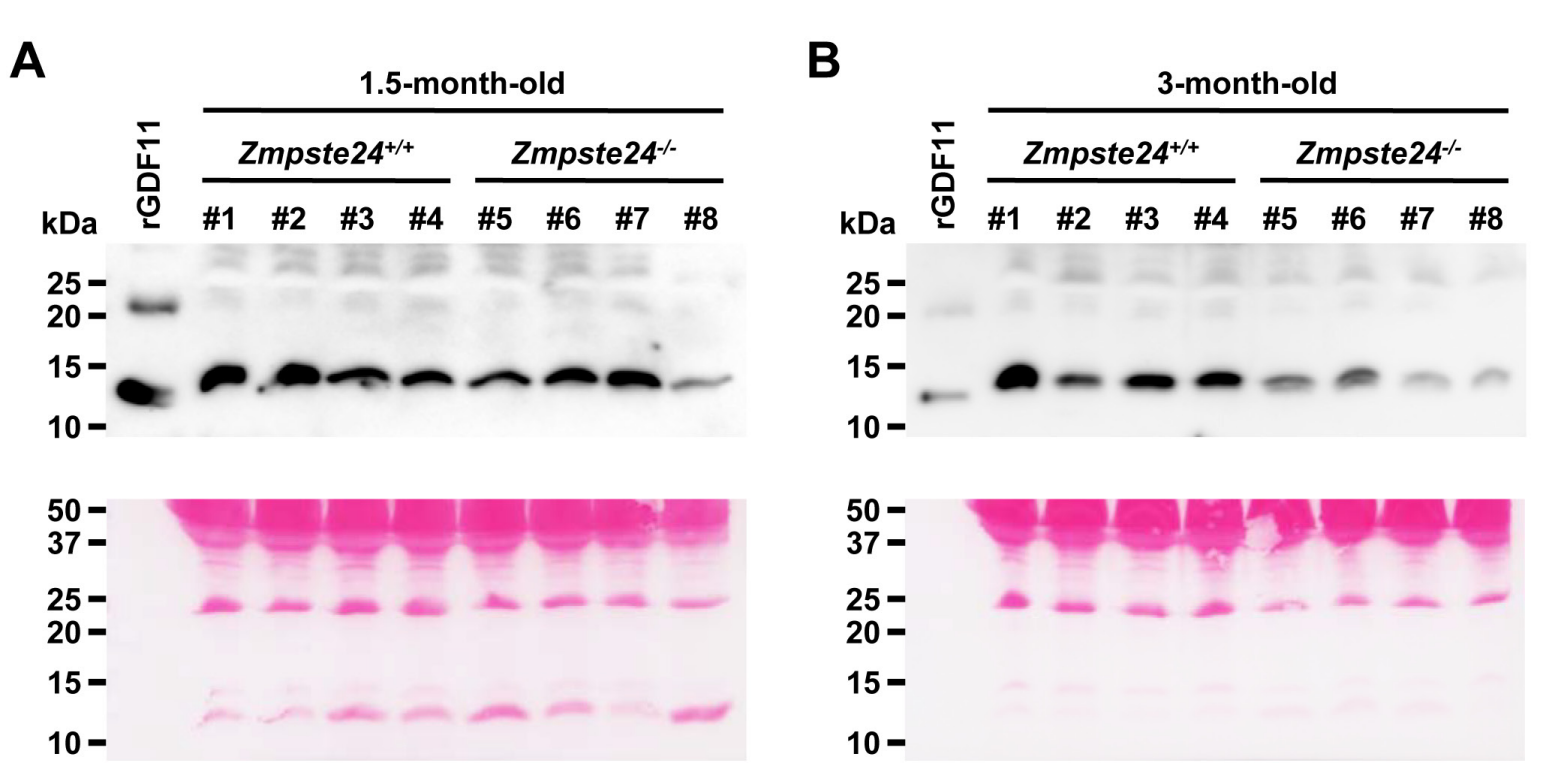
Progeroid *Zmpste24*^−/−^ mice show reduced GDF11/8 plasma levels Western-blot analysis of GDF11/8 plasma levels of young (1.5-month-old) **A.** and aged (3-month-old) **B.** *Zmpste24*^−/−^ and wild-type mice. Note that plasma samples were obtained from the same individuals at the two time points. 2 ng of rGDF11 were loaded as a positive control. Ponceau S stained membranes of the corresponding western-blots are shown to demonstrate equivalent loading (bottom). # indicates mouse number.

To evaluate whether all attributed anti-aging properties of GDF11 may have an overall effect on longevity, we first determined whether GDF11 levels decline in our mouse model of premature aging in the same manner as it has been reported in physiological aging [6, 8]. We performed western-blot analyses with plasma samples obtained from the same wild-type and *Zmpste24*^−/−^ mice at the age of 1.5 months and 3 months, to monitor a possible decline over time, considering that average lifespan of these mutant mice is 4 months and that accelerated aging symptoms start to manifest around the age of 2 months. We used the same commercial antibody as the one previously reported in the original study by Loffredo et al., where GDF11 was first identified as an anti-aging factor [6]. Importantly, recent findings demonstrated that this antibody also recognizes GDF8 (myostatin), a closely related member of the TGF-β superfamily that shares 89% identity in amino acid sequence in the mature active form, questioning those previous published data that showed a GDF11 decline with age [16]. However, to date, no alternative reliable assay capable of detecting endogenous GDF11 in mouse serum has been described [16, 17]. On the basis of these premises, we observed a marked decrease in GDF11/8 plasma levels in *Zmpste24*^−/−^ mice compared with wild-type littermates at the age of 3 months. Interestingly, no significant differences were found when analyzing plasma samples that had been obtained from the same individuals 1.5 months earlier, prior to the development of any aging phenotype (Figure 1A-1B). Ponceau S staining from the corresponding western-blot showed equivalent loading in all lanes. Altogether, these results indicate that the reduction in GDF11/8 blood levels observed in *Zmpste24*^−/−^ *versus* wild-type mice occurs upon the manifestation of the progeroid phenotype.

**Figure 2:**
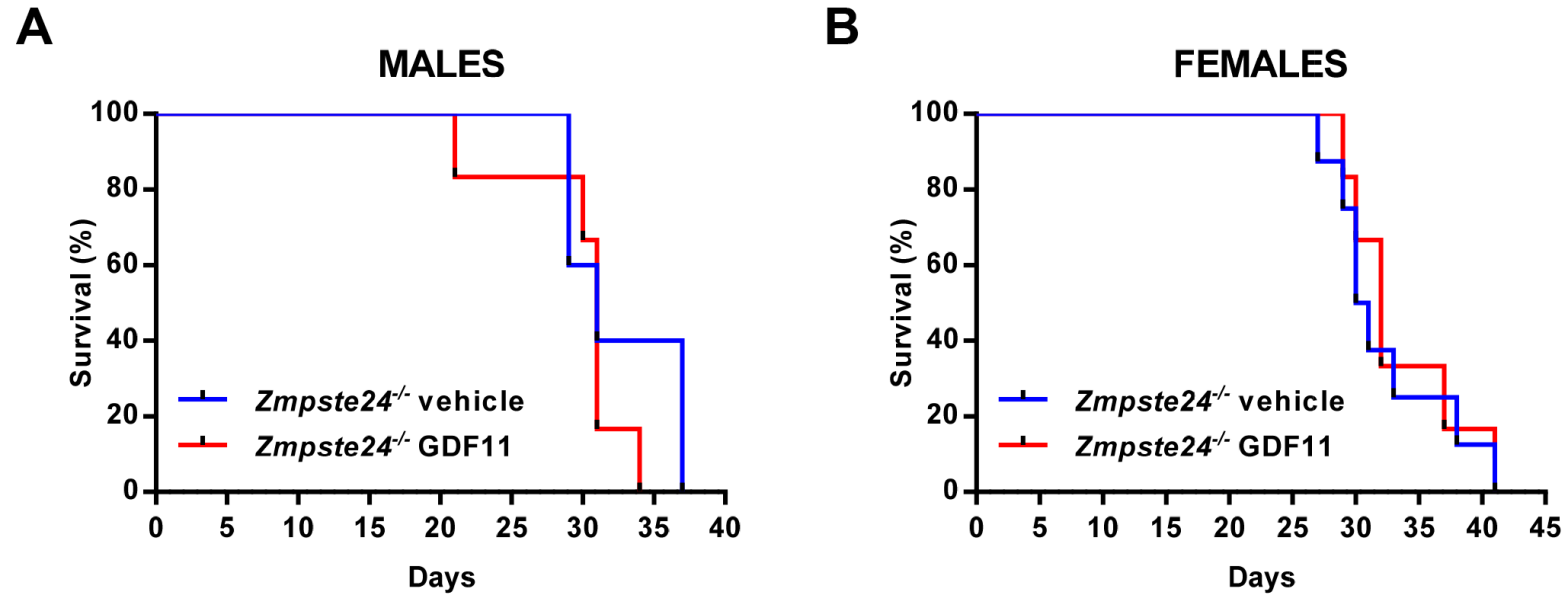
rGDF11 therapy does not extend longevity of *Zmpste24*^−/−^ mice **A.** Kaplan-Meier survival plot of rGDF11-treated (*n* = 5) and vehicle-treated (*n* = 6) male *Zmpste24*^−/−^ mice. **B.** Kaplan-Meier survival plot of rGDF11-treated (*n* = 8) and vehicle-treated (*n* = 6) female *Zmpste24*^−/−^ mice. Mice received a daily intraperitoneal dose of rGDF11 (0.1 mg/kg) or vehicle.

We next evaluated whether GDF11 could be one of the circulating factors capable of slowing down the aging symptoms and extending the lifespan of *Zmpste24*^−/−^ mice, considering its overall effect on a number of aged tissues and the above-mentioned observations (Figure 1B). To test this hypothesis, we did use the same commercial recombinant GDF11 protein (PreproTech) and at the same dosage (0.1 mg/kg, daily) that had been reported to have an anti-aging effect [5-7]. By using this approach, we were able to detect by western-blot an increase in circulating GDF11/8 plasma levels in progeroid *Zmpste24*^−/−^ mice 1 to 2 hours after rGDF11 injection (Figure S1), similar to what it had previously been described (see Loffredo et al. Figure S5). Even though the used antibody was able to recognize both GDF11 and GDF8, it was reasonable to speculate that the protein increase we observed in the blood of the same individual within a 1-2 hour temporal window corresponded to rGDF11. Moreover, alternative detection methods have also demonstrated that daily intraperitoneal injection of rGDF11 (0.1 mg/kg) increased circulating levels of GDF11 above endogenous plasma levels, which were below the detection limit of the assay [17]. Therefore, considering that our goal was to restore progeroid GDF11 plasma levels to a wild-type condition, we were confident that this dose was adequate to test our hypothesis. Thus, male and female *Zmpste24*^−/−^ mice received a daily intraperitoneal injection of rGDF11 (0.1 mg/kg) or vehicle, starting at the age of 2.5 months, once the accelerated aging symptoms started to manifest. rGDF11 treatment did not increase survival of *Zmpste24*^−/−^ mice compared with vehicle-treated animals (Figure 2A-2B). These results were in line with recent reports showing no effect of GDF11 on cardiac or skeletal muscle function, arguing against the “rejuvenating” potential of this protein [16-19].

**Figure 3:**
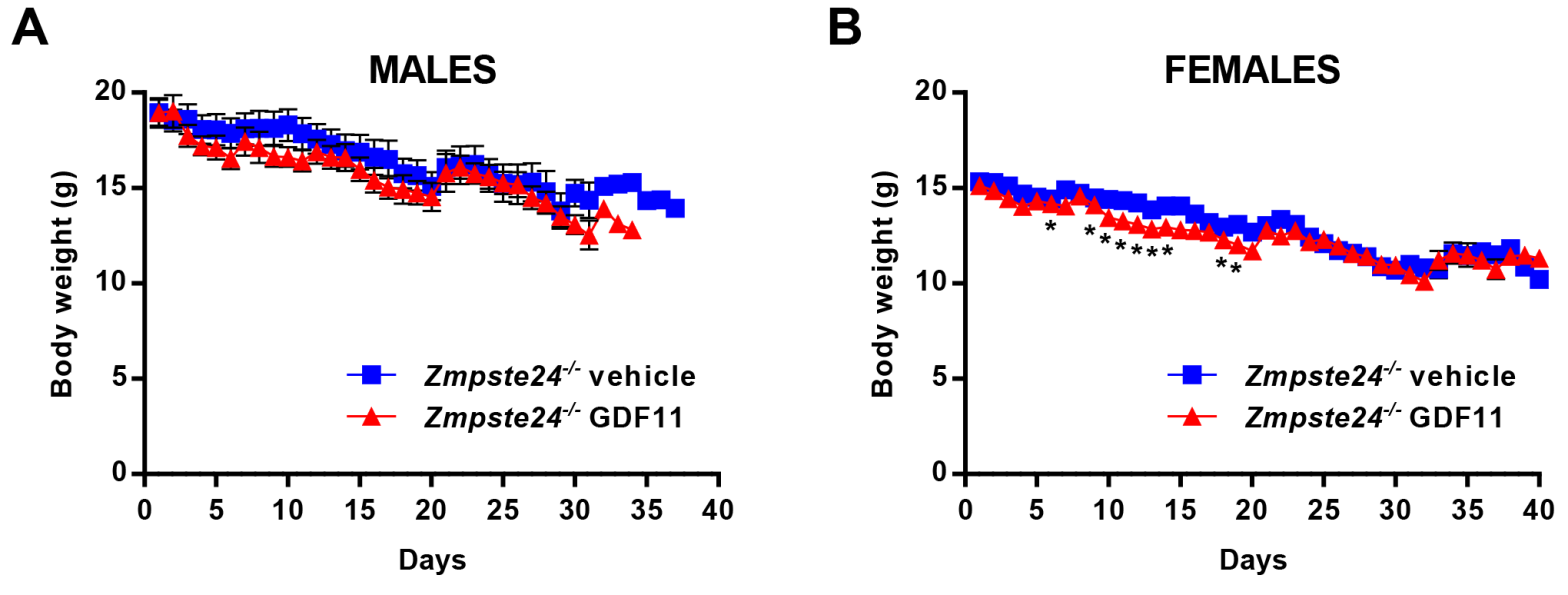
rGDF11 therapy does not prevent body weight loss of *Zmpste24*^−/−^mice Body weights of rGDF11- and vehicle-treated male **A.** and female **B.** *Zmpste24*^−/−^ mice. Data are mean ± SEM. *P*-values were calculated by Student's *t*-test (*P ≤ 0.05).

Additionally, it has recently been suggested that high doses (0.5 mg/kg) of rGDF11 may decrease body weight [20]. In our experiment, we observed that long-term rGDF11 administration at a lower dose (0.1 mg/kg, daily) did not dramatically decrease the body weight of *Zmpste24*^−/−^-treated mice compared with vehicle-treated littermates, although the average body weight was slightly lower in rGDF11-treated mice, being this difference statistically significant at some points of the treatment period in female mice (Figure 3A-3B).

## DISCUSSION

Studies based on heterochronic parabiosis, where a young and an old individual share a circulatory system, have demonstrated that certain age-associated dysfunctions may be rescued or ameliorated by the exposure to a young systemic environment. Interestingly, even the earliest studies using this technique provided evidence of lifespan extension of the older parabiont [21, 22]. Current findings have supported the notion that this beneficial effect has its basis on the restoration of the regenerative capacity of the stem cell pool in the old individual, which suggests that boosting tissue regeneration may slow down organismal aging [23]. In the search for those circulatory factors that decline with age and that are responsible for the maintenance of stem cell performance over time, GDF11 has been identified as a powerful anti-aging candidate with a broad effect on a number of tissues, including cardiac and skeletal muscle and the cerebral vasculature [5-7]. This apparent pleiotropy prompted us to hypothesize that this factor might contribute to extend lifespan, considering the number of key tissues/organs that could benefit. To test this hypothesis, we used a murine model of accelerated aging (Zmpste24-deficient mice), given that it shares most of the features occurring in natural aging [12]. In particular, it has been reported that many of the alterations observed in these progeroid mice are caused by an impaired stem cell function [13, 24, 25]. Moreover, we have previously demonstrated that the coexistence of Zmpste24-deficient cells and Zmpste24-proficient cells (mosaic mice) completely prevented the development of the premature aging phenotype, suggesting that cell-extrinsic mechanisms exerted by the “healthy” cells were responsible for the full reversion of progeroid features [15].

Nevertheless, in addition to the above-mentioned data, to evaluate the possibility that our mouse model of premature aging was suitable to test the effect of GDF11 on longevity, we first determined whether GDF11 levels decline with age in *Zmpste24*^−/−^ mice. Unfortunately, recent studies have reported that current immunoreagents, due to the high similarity between GDF11 and GDF8 (myostatin) amino acid sequences, cannot discriminate between these two closely related members of the TGF-β superfamily in plasma samples [16]. In the light of these findings, the original papers have recently been revisited [20]. Therefore, consistent with what it has now been reported to occur in natural aging, we only observed a marked decrease in the circulating pool of GDF11/8 proteins in *Zmpste24*^−/−^ compared with wild-type littermates when the aging symptoms had already started to manifest in these mice. Thus, no differences where observed when comparing plasma samples from the same individuals obtained at an early age, prior to the development of the progeroid phenotype. These results further support the similarities between our mouse model of premature aging and the process of physiological aging. However, in addition to the criticisms raised about the original observation of a GDF11 decline in aged mice, recent studies have also questioned the capacity of this circulating factor to reverse cardiac and skeletal age-related tissue dysfunction [16-19]. Dosing and the source of the recombinant protein have been claimed as possible factors to explain the discrepancies between the data obtained by different investigators [20, 26]. In this regard, to test our hypothesis about a possible role for GDF11 on lifespan extension, we did use the same commercial rGDF11 protein that has been used in those studies describing its anti-aging properties, and at a dosage capable of raising its levels in *Zmpste24*^−/−^ plasma samples. However, rGDF11 daily treatment did not extend the lifespan of progeroid mice compared with vehicle-treated *Zmpste24*^−/−^ littermates. It has been suggested that some of the original conclusions about GDF11 cardioprotective effects could be due to the decrease in body weight observed as a secondary effect of rGDF11 daily administration [17, 20, 26]. Our results showed that rGDF11 treatment only caused a slightly reduction in the body weight of female *Zmpste24*^−/−^ mice compared with vehicle-treated littermates during the first days of the experiment, whereas no significant differences were observed in the male cohort.

In conclusion, our results demonstrate that circulating GDF11/8 levels are reduced in our mouse model of premature aging, which shares most of the symptoms that occur in normal aging. However, GDF11 protein administration is not sufficient to extend longevity in these progeroid mice. Although accelerated-aging mouse models can serve as powerful tools to test and develop anti-aging therapies common to both physiological and pathological aging, the existence of certain differences between the two processes implies that further investigation is still required to determine whether long-term GDF11 administration has a pro-survival effect on normal aged animals.

## MATERIALS AND METHODS

### Animal experiments

*Zmpste24*^−/−^ mice have previously been described [27]. *Zmpste24*^−/−^ mice were backcrossed 10 generations to C57BL/6N background. All procedures involving mice were conducted in accordance with the guidelines of the Committee for Animal Experimentation of the Universidad de Oviedo. Blood was extracted from the facial veins after anaesthetizing mice and collected into EDTA-coated tubes. Blood was centrifuged at 1000 g at 4°C, and the supernatant was stored at −80°C until analysis. Mice were given a daily intraperitoneal injection of either rGDF11 (PeproTech) at 0.1 mg/kg or vehicle.

### Western-blot

3 μl of plasma samples were separated in 14% SDS-PAGE gels and transferred in CAPS buffer (10 mM 3-[Cyclohexylamino]-1-propanesulfonic acid, pH 10.5, and 20% methanol) onto polyvinylidene fluoride (PVDF) membranes (Millipore). Blots were blocked with 3% nonfat dry milk in TBS-T buffer (20 mM Tris-HCl, pH 7.4, 150 mM NaCl, and 0.05% Tween 20) for 1 h at room temperature and incubated overnight at 4°C with 3% nonfat dry milk in TBS-T buffer with 1:500 anti-GDF11/8 antibody (Abcam, cat. #ab124721). Finally, blots were incubated for 2 h at room temperature in 1.5% nonfat dry milk in TBS-T buffer with 1:2000 goat anti-rabbit IgG horseradish peroxidase (Cell Signaling, cat. #7074S), washed and developed with Immobilon Western Chemiluminescent HRP substrate (Millipore). Chemiluminescent images were acquired with a Fujifilm LAS3000 mini apparatus.

### Statistical analysis

Statistical analyses were performed by using GraphPad Prism 6 software. Log-rank (Mantel-Cox) test was used for Kaplan-Meier survival analysis. Student's *t*-test was used for the analysis of body weight differences between mouse cohorts.

## SUPPLEMENTARY MATERIAL FIGURE


