# Supplementary material for: GDF11 administration does not extend lifespan in a mouse model of premature aging
Source: Oncotarget. 2016 Aug 5;7(35):55951–6. doi: 10.18632/oncotarget.11096 (PMC5302888; doi:10.18632/oncotarget.11096)
Supplement: Supplementary file 1 [file oncotarget-07-55951-s001.pdf]

GDF11 administration does not extend lifespan in a mouse model of premature aging

Supplementary Material

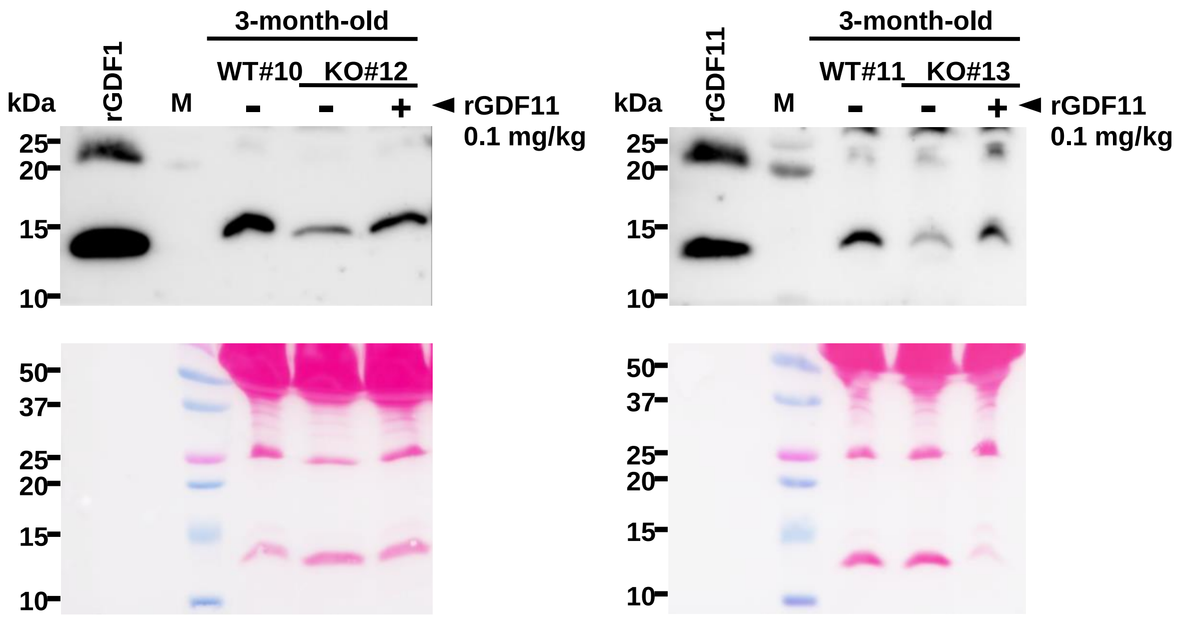

Figure S1. rGDF11 intraperitoneal administration increases GDF11/8 plasma levels in *Zmpste24*$^{-/-}$ mice. GDF11/8 plasma levels of 3-month-old *Zmpste24*$^{-/-}$ mice were evaluated by western-blot analysis before and 2 hours after a single intraperitoneal injection of 0.1 mg/kg of rGDF11. GDF11/8 plasma levels of wild-type littermate mice are shown as a reference. 2 ng of rGDF11 loaded as a positive control. Ponceau S stained membranes of the corresponding western-blots are shown to demonstrate equivalent loading (bottom). # indicates mouse number. M indicates molecular weight marker.
